# Acetylsalicylic Acid and Mood Disorders: A Systematic Review

**DOI:** 10.3390/ph16010067

**Published:** 2022-12-31

**Authors:** Monika Dominiak, Adam Gędek, Michalina Sikorska, Paweł Mierzejewski, Marcin Wojnar, Anna Z. Antosik-Wójcińska

**Affiliations:** 1 Department of Pharmacology, Institute of Psychiatry and Neurology, Sobieskiego 9, 02-957 Warsaw, Poland; 2 Praski Hospital, Aleja Solidarności 67, 03-401 Warsaw, Poland; 3 Medical Center of Postgraduate Education, Medical University of Warsaw, Żwirki i Wigury 61, 02-091 Warsaw, Poland; 4 Department of Psychiatry, Medical University of Warsaw, Nowowiejska 27, 00-665 Warsaw, Poland; 5 Department of Psychiatry Faculty of Medicine, Collegium Medicum, Cardinal Wyszynski University in Warsaw, Woycickiego 1/3, 01-938 Warsaw, Poland

**Keywords:** acetylsalicylic acid, aspirin, ASA, mood disorders, bipolar disorder, major depression, depressive episode, mania, animal model of mania, animal model of depression

## Abstract

The effects of acetylsalicylic acid (ASA) on mood disorders (MD) and on inflammatory parameters in preclinical and clinical studies have not yet been comprehensively evaluated. The aim of this study was to systematically summarize the available knowledge on this topic according to PRISMA guidelines. Data from preclinical and clinical studies were analyzed, considering the safety and efficacy of ASA in the treatment of MD and the correlation of inflammatory parameters with the effect of ASA treatment. Twenty-one studies were included. Both preclinical and clinical studies found evidence indicating the safety and efficacy of low-dose ASA in the treatment of all types of affective episodes in MD. Observational studies have indicated a reduced risk of all types of affective episodes in chronic low-dose ASA users (HR 0.92, 95% CI: 0.88, 0.95, *p* < 0.0001). An association between ASA response and inflammatory parameters was found in preclinical studies, but this was not confirmed in clinical trials. Further long-term clinical trials evaluating the safety and efficacy of ASA in recurrent MD, as well as assessing the linkage of ASA treatment with inflammatory phenotype and cytokines, are required. There is also a need for preclinical studies to understand the exact mechanism of action of ASA in MD.

## 1. Introduction

Mood disorders are severe mental illnesses characterized by periods of depression in major depressive disorder (MDD) or depression and other mood episodes such as hypomania/mania or mixed state in bipolar disorder (BD). A lifetime prevalence of MDD is estimated at 2 to 20%, while BD affects round 2% of people worldwide, and their burden has been increasing in recent years [1,2,3]. These disorders are associated with a significant impairment in quality of life, increased suicide risk, and 8–14 years reduction in life expectancy compared to the general population [4,5,6,7,8]. According to the current medical knowledge, antidepressants, mood stabilizers, as well as biological treatments such as electroconvulsive therapy, are used in the treatment of mood disorders. Nevertheless, currently available treatment, even when correctly applied and with satisfactory patient compliance, is insufficiently effective in up to one third of the cases. Treatment-resistant depression is often recognized in this group of patients (about 30%). They experience symptoms and impaired psychosocial functioning for about half of their lives. Hence, there is a need for novel, more effective therapies.

The search for such therapies, both pharmacological and non-pharmacological, has been ongoing for years. Nevertheless, no game-changing treatment for BD has been seen for over two decades. One possible explanation could be connected to mechanisms underlying these conditions that are still undiscovered. This fact greatly impacts the introduction of targeted treatment. It has been suggested that the pathogenesis may be related to disturbances in biological rhythms, inflammation in the central nervous system, or dysfunction in the immune system. In recent years, with the advancement of immunopsychiatry, an inflammatory background of mood disorders has been emphasized [9,10,11,12]. Mood disorders are significantly more common in patients with autoimmune diseases [13,14]. A higher prevalence of comorbid disorders, such as autoimmune disease, obesity, diabetes, dyslipidemia, or cardiovascular disease, is also observed in patients with mood disorders [15,16]. Inflammatory markers have been also found to be elevated in patients with mood disorders, both peripherally and centrally. Depressed individuals have elevated levels of interleukin 6 (IL-6), tumor necrosis factor alpha (TNF-alpha), IL-10, IL-13, IL-18, IL-12, the soluble IL-2 receptor, and the soluble TNF receptor 2 as compared to healthy controls [17,18]. Furthermore, the response to antidepressant treatment in MDD may be related to inflammation markers, such as baseline levels of IL-8, IL-6, and C-reactive protein (CRP) levels [19,20]. Despite some differences in the results obtained for bipolar disorder, the concentration of substances, such as TNF-alpha, the soluble TNF receptor 1, the IL-1 beta receptor, the soluble IL-2 receptor, and the soluble IL-6 receptor, seems to change during the course of the illness [12]. The expression patterns of cytokines depend on the stage and phase of BD. Probably, they are linked to low-grade inflammation maintenance and become elevated in conditions of acute inflammation, such as mania or depression. According to recent meta-analyses, CRP and TNF appeared to be potential state markers for BD, while IL-6 seemed to represent a trait marker [21]. However, it is unclear whether inflammation ceases and immune markers normalize in the euthymic state or whether the inflammatory process continues during this state [22]. Furthermore, several studies have pointed to signs of inflammation in postmortem brain sections of patients with mood disorders, although, again, the results are not homogeneous [23,24]. The brains of patients with mood disorder are characterized by an excessive activation with an inflammatory phenotype [10,23,25,26]. Neuroimaging has also confirmed these findings [27,28]. In addition, inflammation may also be influenced by the hypothalamic–pituitary–adrenal axis or by altered gut microbiota, which require further investigation in patients with mood disorders [10,11,29]. As a result of discovering the role of inflammation in mood disorders, several anti-inflammatory preparations have been explored. Although the results are inconclusive, many studies report that certain drugs can positively affect the clinical condition of patients with mood disorders, including acetylsalicylic acid (commonly known as aspirin), celecoxib, n-acetylcysteine, infliximab, and minocycline [30,31]. The link between mood disorder and inflammation is certain, but the exact mechanisms remain unclear. There is a growing need to answer the question of the effects of anti-inflammatory drugs on the course of mood disorders.

One of the most commonly prescribed drugs in medical practice is aspirin (ASA), which belongs to the category of non-steroidal anti-inflammatory drugs (NSAID). The dosage of ASA can range from 300 mg to 1200 mg for acute analgesia or chronic inflammatory disorders, and it can be administered at low dosages within the range of 30–162 mg daily for cardiovascular and cerebrovascular disease prevention [32,33]. The drug is also beneficial for cancer patients such as ovarian, hepatocellular, prostate, or breast cancer and reduces the risk of colon cancer [34,35,36]. Population-based studies conducted in recent years have suggested that ASA may also be beneficial in treating neuropsychiatric conditions, including mood disorders [37,38,39,40]. ASA irreversibly inhibits cyclooxygenase-1 (COX-1) and cyclooxygenase-2 (COX-2) enzymes involved in neuroinflammation [41,42]. Through inhibition of the arachidonic acid pathway, the drug reduces prostaglandin E2 as well as pro-inflammatory biomarkers such as IL-1beta and TNF-alpha, and oxidative stress biomarkers. ASA has been reported to have neuroprotective properties in recent studies, but the exact mechanism of action is still unknown. Peroxisome proliferator-activated receptors (PPAR) alpha in astrocyte and microglia cells can be regulated by ASA, which affects neurotrophic factors production, synaptic plasticity, and amyloid plaque removal. An anti-inflammatory molecule called IL-1Ra may play a role in this process [43]. Furthermore, ASA can also promote oligodendroglial differentiation and activate AMP kinase in central nervous system (CNS) cells [44,45]. Aspirin’s neuroprotective, pro-cognitive, and antidepressive effects may be supported by any of these mechanisms. Despite the lack of a clear mechanism for the effects of ASA on neuroinflammation, preclinical, observational, and a few intervention studies indicate its potential as a neuropsychiatric drug. Aside from its potential efficacy, the repurposing of ASA is particularly attractive since it is an old and well studied drug on the World Health Organization Model List of Essential Medicines [46]. Additionally, aspirin wholesale costs in developing countries are estimated at 0.002–0.025$, as of 2014 [47]. The use of ASA as an adjunctive treatment for mood disorders could therefore benefit patients worldwide if effective.

The aim of this review was to systematically summarize the available knowledge on the use of ASA in mood disorders. We have analyzed the results of preclinical and clinical (observational and interventional) studies, distinguishing between: (1) the effects of ASA on affective symptoms in depression; (2) bipolar disorder; (3) the adverse effects of ASA therapy as an add-on treatment to antidepressants or mood stabilizers; and, finally, (4), the correlation of inflammatory parameters with the effect of ASA treatment. To our knowledge, such a comprehensive review has not been conducted to date.

## 2. Materials and Methods

This systematic review was conducted according to the PRISMA statement (Preferred reporting items for systematic review and meta-analyses [48]).

### 2.1. Eligibility Criteria

We used PICO framework-based research question for this review (Table 1). If articles met predefined criteria, they were included and categorized as interventional, observational, or preclinical studies.

The inclusion criteria were as follows: (1) preclinical, observational, or interventional studies of any design; (2) concerning mood disorders or animal model of mood disorders; (3) study on the effect of acetylsalicylic acid (ASA) on mood disorders or for behavioral testing in an animal model of mood disorders; (4) participants over 18 years of age and under 65 years of age (applies to clinical studies); (5) published in English.

Articles were excluded if they were not original articles or had a different study design, were not in English, the full text was not available, were not published, or did not conform to PICO.

### 2.2. Information Sources, Search Strategy and Selection Process

PubMed, PsycInfo, and Embase databases were searched (from earliest to current) to identify suitable articles. In August 2022, three independent reviewers (AG, MS, MD) performed a search of the target online databases for eligible studies. The search string used in was (“bipolar disorder” or “bipolar depression” or “mania” or “hypomania” or “mixed episode” or “major depression” or “mood disorders”) and (“aspirin” or “acetylsalicylic acid” or “ASA”). Follow-up citations were also scanned for relevant articles. Additionally, we performed search among the grey literature sources (ProQuest Dissertations and Theses Online, The Grey Literature Report from the New York Academy of Medicine, and OpenGrey) and in the clinical trials registry. Search results were downloaded into EndNote version X9. No filters were applied during the search. Three reviewers (AG, MS, MD) independently identified potentially relevant articles, removed duplicates, and then reviewed titles and abstracts. To access eligibility criteria, the full text of all qualified or uncertain studies was obtained. Any disagreements between reviewers were resolved via discussion to achieve consensus.

### 2.3. Data Collection Process

The literature was retrieved, selected, and extracted independently by three researchers (AG, MD, MS). Conflicting information was discussed to reach an agreement and, if necessary, the third investigator was consulted.

The following data were extracted: authors, year of publication, country, study design, sample and control sample size, duration, time of study, characteristic of the research and control group (sex, mean age, diagnosis, treatment), dose of ASA, and outcomes (in preclinical studies—behavioral test, histological and biochemical analysis, in clinical studies—the incidence of affective episodes—hazard ratio, incident density, odds ratio, the effect of the intervention on affective symptoms as measured by clinical scales, reports on adverse effects).

### 2.4. Study Risk of Bias Assessment

The risk of bias for randomized trials was evaluated using the evaluation method recommended by Cochrane System Reviewer Manual 5.1 (a revised tool to assess risk of bias in randomized trials-RoB 2), including sequence generation, allocation concealment, blinding, missing outcome data, selective reporting, and other bias [49,50]. To assess the risk of bias in non-randomized studies with intervention we used ROBINS-I tool (Risk Of Bias In Non-randomized Studies of Interventions) [51], and in observational studies ROBINS-E tool (The Risk Of Bias In Non-randomized Studies of Exposure) [52]. At least two independent reviewers assessed the risk of bias for each study. Conflicting information was discussed to reach an agreement and, if necessary, the third investigator was consulted. The Robvis tool was used to illustrate the results [53].

### 2.5. Data Synthesis and Analysis

Search results from Endnote X9 have been transferred to RevMan5. In this review, studies were divided into categories based on study design: preclinical, observational, or interventional studies. Heterogeneity was evaluated visually on the forest plot and statistically using the chi square, I^2^ and tau^2^ methods. Whenever the I^2^ test was below 90% (substantial heterogeneity), the results were pooled. Although a meta-analysis was originally planned, it was abandoned due to high heterogeneity of the data, especially regarding interventional trials. Only observational trials have showed a substantial heterogeneity and were further evaluated by using random effect model. The results of each category of study design were described and summarized.

## 3. Results

### 3.1. Study Characteristics

**Figure 1:**
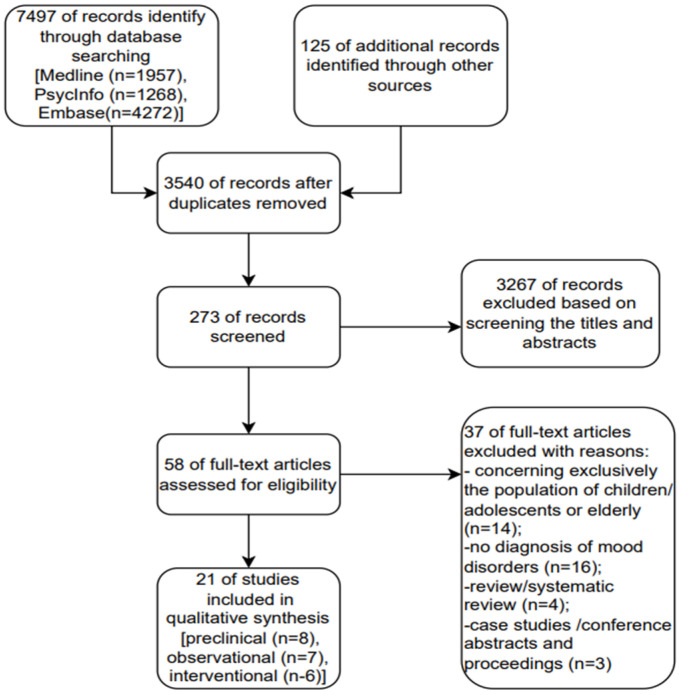
Flow chart: an overview of the study selection process.

The search strategy identified 7497 abstracts. After a review of the titles and abstracts, a total of 273 potentially relevant articles were chosen, of which 58 were qualified for full-text reading and were evaluated using the inclusion and exclusion criteria. Overall, 21 studies met the inclusion criteria. A flowchart of the review process is plotted in Figure 1.

The included studies were published between the years 2006 and 2022. Among the identified studies, eight were preclinical (animal research) and 13 were clinical trials (seven observational and six interventional). All preclinical studies involved rodents and used behavioral tests as models of anxiety, depression, or mania to assess the effects of ASA. Regarding clinical trials, a total of 13 studies were identified that described the efficacy of ASA for mood disorders in the adult population (18–65 years). Among the seven observational studies, one was prospective, with a mean follow-up duration of 5.2 years [54], and six were retrospective with a mean duration of 8.8 years (range 5–10 years) [37,38,39,40,55,56]. In addition, two of the studies consisted of a controlled study and a retrospective study [37,55]. The extracted data from observational studies represent a sample of 637,000 patients. As for the identified interventional clinical trials evaluating the efficacy of ASA in mood disorders, four were double-blind RCTs [57,58,59,60], one was open-label RCT [61], and one was a non-randomized open-label pilot study [62]. The average study time was 24 weeks (range 4–52 weeks), and the total patient sample was 317.

The majority of studies were conducted in the USA (4), followed by China (3), Australia (2), Belgium (2), Denmark (2), Iran (2), Israel (2), and Italy (2), with single representations from Switzerland (1) and the Netherlands (1).

### 3.2. Quality Assessment

**Figure 2:**
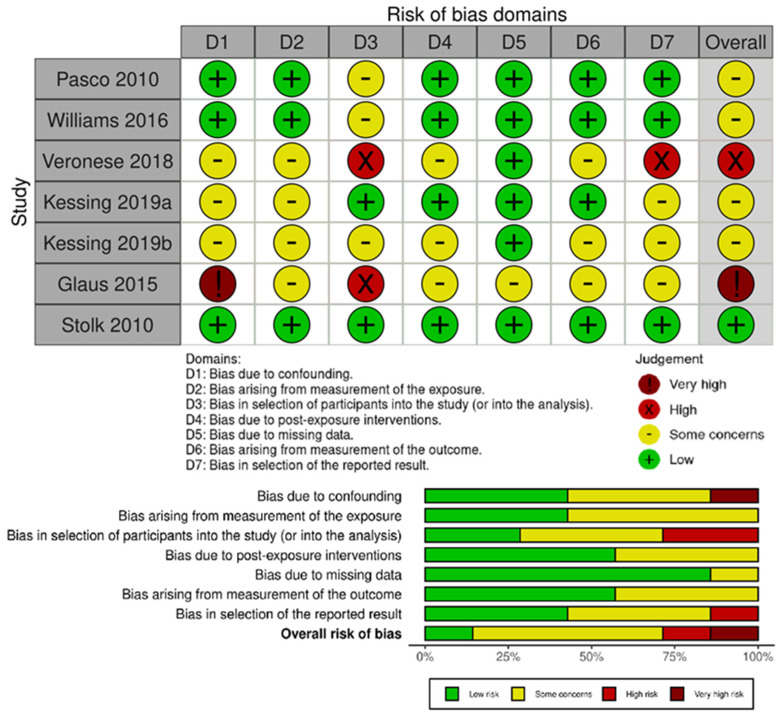
Risk of bias for observational studies separately and summarizing with ROBINS-E tool.

**Figure 3:**
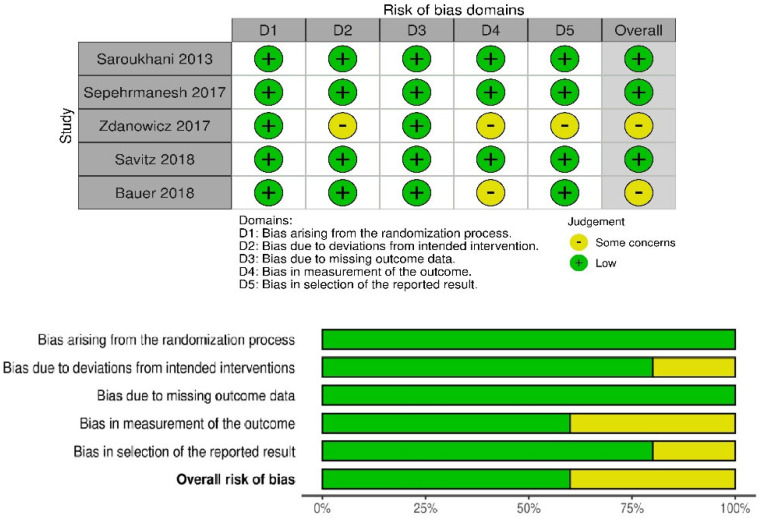
Risk of bias for interventional randomized studies separately and summarizing with Rob2 tool.

**Figure 4:**
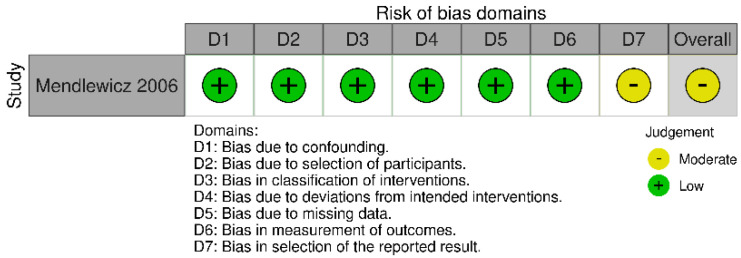
Risk of bias for interventional non-randomized study with ROBINS-I tool.

The observational studies were ranked according to the ROBINS-E tool. One of the six included studies was rated as ‘low risk of bias’ [38], the other four as ‘some concerns’ [37,39,40,55], of the remaining two, one as ‘high risk’ [56] and one as ‘very high risk’ [54]. Interventional studies were assessed according to the RoB 2 tool (a revised tool for assessing Risk of Bias in randomized trials) and ROBINS-I tool (Risk Of Bias In Non-randomized Studies-of Interventions). Of the six interventional studies, three were ranked as ‘low risk of bias’ [57,58,60] and the other two as ‘some concerns’ [59,61]. One non-randomized open-label pilot study was evaluated using the ROBINS-I tool and was assessed as “moderate risk” [62]. Risk of bias for all studies is presented in Figure 2, Figure 3 and Figure 4.

### 3.3. Preclinical Studies

A total of eight in vivo studies were identified (Table 2).

#### 3.3.1. Effects of ASA on Depressive Symptoms in Animal Models

The results of animal studies are ambiguous; some studies indicate that ASA may enhance the antidepressant effect of fluoxetine [63,64,65] or lithium [66] or also has an independent antidepressant effect [67], on the other hand, ASA inhibited the antidepressant effect of citalopram [68].

The results of one study have also indicated that ASA might accelerate the onset of action of SSRIs [64].

Interestingly, the study of Wang et al. has also pointed out that ASA had antidepressant augmentation properties in fluoxetine treatment resistant depressive rats induced by chronic unpredictable mild stress (CUMS) [65]. Adjunctive ASA treatment significantly improved the depressive behaviors and downregulated the COX-2 level and PGE2 concentration in the hippocampus.

The antidepressant effect of ASA alone has been demonstrated in forced swimming test in rats [67]. ASA dose-dependently decreased immobility, without altering the locomotor activity. The effect of ASA was similar to fluoxetine and imipramine.

There are also results indicating that ASA and other anti-inflammatory drugs may suppress antidepressant effect of SSRIs in mice [68]. Ibuprofen reversed citalopram-induced increase in cytokines and p11 protein, and the last one seems to be necessary for SSRI antidepressant activity. This discrepancy may be due to different animal species used (rats vs. mice), or it may be the result of the use of different doses of ASA. In the only study that did not confirm the antidepressant efficacy of ASA, very high doses of ASA were used—210 mg/kg [68].

It has to be noted that studies by Alboni et al. (2018) and Brunello et al. (2006) were based on a chronic escape deficit model of depression [63,64]. This model was first described by Gambarana et al. (2001) [69]. However, this chronic escape deficit test has not been validated and is also not commonly used to study antidepressants. Other studies discussed [65,66,67,68] were based on commonly used tests to study antidepressants, i.e., forced swimming test and tail suspension tests [70]. It is worth emphasizing at this point the difficulty of assessing the quality of preclinical studies and thus the possibility of drawing firm conclusions. The range of ASA doses studied varied widely, ranging from 1 mg/kg–210 mg/kg. Most studies used low doses of ASA in the range of 1–50 mg/kg.

#### 3.3.2. Effects of ASA on Manic Symptoms in Animal Model

The literature review identified only one study that evaluated the issue of manic symptoms in a rat model of mania [71]. In this study, the mania-like state was induced with ouabain. The effect of ouabain administration was assessed by increased locomotor activity, which was reversed with ASA. The authors of this study conclude that ASA can be used as a promising adjunctive therapy in the treatment of bipolar disorder.

#### 3.3.3. Adverse Effects of ASA in Rodents

Only one study addressed the safety of ASA therapy in rodents [72]. Authors studied the safety of combining lithium and ASA. They conclude that co-administration of low-dose ASA (1 mg/kg) with low-dose lithium mitigates the typical renal side-effects of standard-dose lithium.

#### 3.3.4. Effects of ASA on Inflammatory Parameters in Rodents

The effects of ASA on peripheral and central inflammatory parameters were evaluated in four studies [65,66,67,71]. These studies monitored plasma levels of inflammatory parameters, such as IL-6, TNF-alpha [66,67,71], CRP, IL-1β, IL-2, IL-10, INF- γ, PGE2, BDNF, TLR3, TLR4 [71], and brain tissue levels of COX-2 and PGE2 [65]. All these studies (4/4) confirmed that ASA enhanced anti-inflammatory effects [65,66,67,71].

### 3.4. Observational Studies

A total of seven observational studies evaluating the effectiveness of chronic ASA use on the occurrence of mood disorders were identified (Table 3).

**Figure 5:**
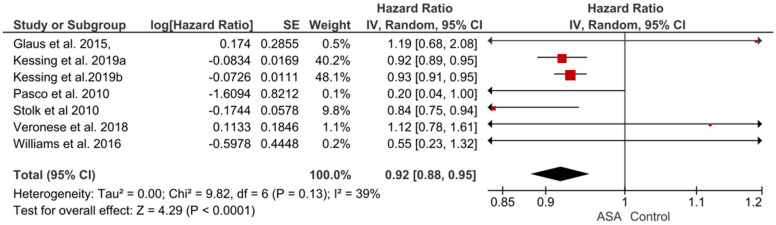
Observational studies on the effect of ASA on the occurrence of mood disorders–the pooled hazard ratio.

Most of these studies (n = 5) addressed the emergence of depression [37,39,54,55,56], and the remaining two assessed the effect of ASA on symptoms of bipolar disorder [38,40]. All the studies assessed the potential effectiveness of ASA in reducing the risk of affective episodes. None of the observational studies, however, addressed the question of safety of therapy, as well as the association between pro- and anti-inflammatory factors and the occurrence of mood disorders. The heterogeneity of all observational studies (addressing both depressive and bipolar disorder symptoms) was substantial (I^2^ = 39%, chi^2^ = 9.82; *p* = 0.13, Tau^2^ = 0.0). The pooled HR was 0.92 [0.88,0.95]. Test for overall effect: Z = 4.29 (*p* < 0.0001) (Figure 5).

All observational studies presented age-adjusted results. However, in four studies, age differed significantly between study groups [37,54,55,56], and, in three studies, no information was provided [38,39,40].

Of the included observational studies, five reported gender-adjusted results [38,39,40,54,56]. In addition, one study was conducted only in a female population [37], while one was conducted only in a male population [55].

Five studies reported results adjusted for comorbidity [38,39,40,54,56]. However, in two studies, the rate of cardiovascular events and diabetes was higher in the ASA group [54,56].

#### 3.4.1. Observational Studies–Effects of ASA on Depressive Episodes

**Figure 6:**
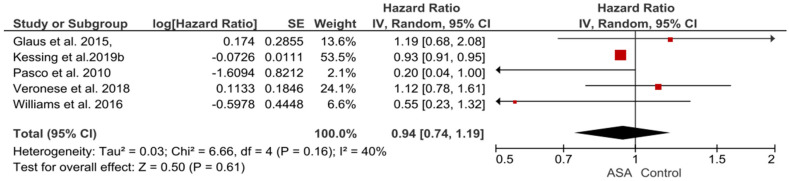
Observational studies on the effect of ASA on the incidence of depression—the pooled Hazard Ratio.

As for the study on the effect of ASA on the incidence of depression, it included both populations—those with no prior history of depression and those with a positive history of depression. The control groups were the country’s healthy population. Three out of five (60%) of these studies indicate a reduced risk of a depressive episode in chronic ASA users compared to the control group over a long-term time horizon of 7–10 years [37,39,55]. In all studies, patients took ASA for cardiac/neurological indications at a low dose as thromboprophylaxis, but only one study specified a dose range-75–150 mg [39]. In contrast to previous studies, the authors of two other studies found no reduced risk of depression [54,56]. As in the three studies above, here similarly, patients received low-dose ASA for thromboprophylaxis indications for a similarly long time. It is worth noting that one of these two studies was prospective (mean follow-up of 5.2 years) and included 132 patients. However, the authors of the study emphasize that the study and control groups differed significantly in terms of age (mean age was higher in the study group) and the prevalence of overweight, diabetes, hypertension, and heart disease—all of which were significantly more common in the study group. The results of this study should therefore be considered with this limitation in mind. It is also worth mentioning that this study was evaluated as a ‘very high risk’ [54]. The heterogeneity of studies evaluating effect of ASA on depressive episodes was substantial (I^2^ = 40%, chi^2^ = 6.66; *p* = 0.16, Tau^2^ = 0.03), test for overall effect: Z = 0.5 (*p* = 0.61) (Figure 6).

#### 3.4.2. Observational Studies-Effects of ASA on Bipolar Disorder

**Figure 7:**
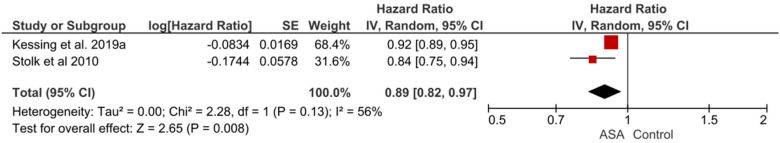
Observational studies on the effect of ASA on bipolar disorder symptoms–the pooled hazard ratio.

Two good quality studies have provided data on the efficacy of chronic ASA use on symptoms of bipolar disorder. Both studies (2/2) indicate the potential effectiveness of ASA in reducing the risk of affective episodes over a 10-year follow-up period. A study by Stolk et al. (2010) showed that chronic use of low doses of ASA (up to 80 mg/day) improves the course of illness in patients with BD [38]. Equally interesting results are provided by a study in patients without a prior diagnosis of BD [40]. Chronic treatment with low-dose ASA (75–150 mg/d) was associated with a reduced risk of experiencing an episode of mania, another affective episode in BD, or taking lithium compared to the control group. The heterogeneity of studies evaluating effect of ASA on bipolar disorder symptoms was substantial (I^2^ = 56%, chi^2^ = 2.28; *p* = 0.13, Tau^2^ = 0.0). The pooled HR was 0.89 [0.82,0.97]. Test for overall effect: Z = 2.65 (*p* = 0.008) (Figure 7).

### 3.5. Interventional Studies

We identified six interventional studies that evaluated the effect of ASA on affective symptoms (Table 4). All the studies were of good quality (‘low risk of bias’/‘some concerns’). Four studies focused on the patient population with bipolar disorder (three RCTs and one non-randomized open pilot study) [57,59,60,62], and three studies focused on depression (one double-blind RCT, one open RCT, and one non-randomized open pilot study) [58,61,62]. The latter study included a depressive episode in the course of both bipolar disorder and recurrent depressive disorder. Due to the high heterogeneity of the study measures, a pooled average for the intervention studies was not calculated.

The mean age of patients treated with ASA ranged from 35.9 ± 9.0 to 49 ± 15.2 [57,59]. In four of the five studies, the placebo groups did not differ significantly in this aspect at the start of the study [57,58,59,60] and ranged from 34.25 to 47.8 ± 7.3 [58,61]. The ASA group in one study were significantly older than the placebo group. However, there was no correlation between age and HAMD scale, except for one month of the study [61].

There was no significant difference in the sex ratio between the ASA group and other groups in any of the studies. The distribution of the ASA and placebo group is not reported in one study [61]. In one case, only men were involved [57].

Most studies did not report differences between study groups in the frequency of comorbidities. The group taking ASA had significantly less CNS disease than the other groups in the Savitz et al. study [60].

SSRIs were used as the main therapy in two studies [58,62], and SSRIs with SNRIs were used in another study [61]. Patients used different therapies in two studies, but the differences were not statistically significant between research groups [59,60]. In one study, patients were treated with lithium [57].

#### 3.5.1. Interventional Studies-Effects of ASA on Depressive Episodes

Of the three studies identified, two were placebo-controlled and used ASA as an add-on treatment to an antidepressant [58,61]. One study—a non-randomized open pilot study—assessed treatment response and remission in patients with bipolar disorder and recurrent depressive disorder previously treated with SSRIs who did not respond to treatment [62]. Study durations and study sample ranged from four weeks (n = 21) [62], to eight weeks (n = 100) [58] to two years (n = 40) [61]. All these studies used low doses of ASA, ranging from 100–160 mg/day.

Of the three studies evaluating the effect of ASA on a depressive episode, two (66%) reported the potential efficacy of ASA in the treatment of depression [58,62]. A study by Zdanowicz et al. (2017) found no difference between the ASA and placebo groups in terms of depressive episodes. However, it is worth noting the good tolerability of ASA used at a dose of 100 mg for two years was found in this study. Additionally, the SNRI + ASA group achieved a better response than the SSRI + ASA. However, as the authors suggest, this was not necessarily related to a better response, but a different baseline condition of the patients, related to various factors, for example BDNF levels [61].

There was no evidence that age, sex ratio, mental comorbidities, or medication used affected treatment efficacy.

#### 3.5.2. Interventional Studies—Effects of ASA on Bipolar Depression

Of the four studies analyzing the effect of ASA on the BD patient population, three investigated the effectiveness of this intervention in reducing depression [59,60,62]. In contrast, one study did not evaluate the effect of ASA on affective episodes in bipolar disorder, but only on sexual function in lithium-treated patients [57]. The latter study involved only male participants.

Study durations ranged from four weeks [62] to 6 weeks [57,60] to 16 weeks [59]. The study populations were as follows: N = 21, N = 25, N = 32 and N = 99 (57, 59, 60, 62, respectively). The dose of ASA was in the range of 160–1000 mg/day. In all studies investigating the efficacy of ASA in bipolar depression, 100% (3/3) confirmed the efficacy of ASA, noting that in one [59], this was only the case for ASA in combination with N-acetylcysteine.

There was no evidence that age, sex ratio, mental comorbidities, or medication used affected treatment efficacy.

#### 3.5.3. Interventional Studies—Adverse Effects of ASA

Four interventional studies addressed, in addition to efficacy, the safety of ASA therapy as an add-on treatment. One study provided data on the safety of the combined treatment of ASA with lithium [57]. It was observed that such therapy was well tolerated and did not affect lithium levels. Similarly, the study by Sepehrmanesh et al. (2017) has found that the combination of ASA with sertraline is safe and effective treatment [58]. The safety of ASA therapy was also addressed in a study by Bauer et al. (2018), which used ASA at a very high dose of 1000 mg/d for 16 weeks [59]. One of the longest interventional studies, lasting two years, also confirmed the safety of ASA as an add-on treatment to antidepressants at a dose of 100 mg per day [61].

There was no evidence of adverse interactions of the drugs used in these studies (SSRI drugs, duloxetine, lithium, anticonvulsants, antidepressants, antipsychotics, benzodiazepines) with ASA. However, data on somatic comorbidities and drugs used in somatic diseases are lacking in these studies.

#### 3.5.4. Interventional Studies-Effects of ASA on Inflammatory Parameters

Two of the six studies investigated the association of inflammatory markers with affective symptoms [59,60]. In contrast to preclinical studies, these studies failed to demonstrate an association between ASA response and IL-6 and C-reactive protein levels [59,60], as well as with urinary thromboxane B2 (11-D-TXB2) levels, serving as an indirect marker of cyclooxygenase (COX) activity [60].

## 4. Discussion

In light of recent reports of pro-inflammatory markers found in patients with mood disorders linking immunology and psychiatry, one of the most commonly prescribed drugs in medical practice, ASA, has begun to be tested in clinical trials as a potentially effective substance in mood disorders [12]. In this review, we evaluated the available studies on the use of ASA for mood disorders in the adult human population and in animal models. One of the first interesting findings was the observation that the typical pathway of preclinical and then clinical studies was not followed in this case. Seven of the eight preclinical studies were a consequence of earlier clinical studies. We found that, with isolated exceptions, observational studies were conducted first, launching preclinical and clinical interventional studies.

The main finding of this systematic review is the evidence supporting the positive effect of ASA on both depressive and other affective episodes in the course of bipolar disorder. In the 2019 systematic review, the pooled hazard ratio from the three cohort studies identified at the time was 0.624 (95% CI: 0.05, 1.198, *p* = 0.033), indicating a reduced risk of depressive episode in ASA-exposed individuals [73]. We identified seven observational studies finding a pooled HR of 0.92 (95% CI:0.88, 0.95, *p* < 0.0001). This result is therefore consistent with the previous review. In addition, it also adds information about the potential efficacy of ASA in reducing the risk not only of depressive episodes, but also of other affective episodes, i.e., manic and mixed episodes in the course of BD.

Regarding depression, we have found evidence in both preclinical and clinical studies indicating the efficacy of ASA. However, this topic seems to have been sparked largely by observational epidemiological studies, and the few preclinical and interventional studies to date have only begun to follow this direction. In clinical studies, ASA was reported to reduce the risk of depression in three of the five observational studies (60%) [37,39,55] and in two of the three (66%) interventional trials [58,62].

The outcomes of the studies for bipolar disorder were far more consistent than for depressive episodes. One hundred per cent of both preclinical and clinical studies have confirmed the beneficial effects of ASA on BD. A preclinical study in an animal model confirmed the efficacy of ASA in reducing manic symptoms [71]. Similarly, in observational studies, ASA was effective in reducing the risk of all episodes in BD and was associated with a lower risk of illness worsening [38,40]. In contrast, interventional studies have investigated the efficacy of ASA only in bipolar depression and confirmed its effectiveness in this indication [59,60,62]. It should be noted, however, that of all the interventional studies evaluating the efficacy of ASA in mood disorders, only two were RCTs with large sample size and had sufficient power to confirm the beneficial effects of ASA [58,60]. Both studies investigated the effect of ASA on the reduction of depressive symptoms–the study of Sepehrmanesh et al. (2017) in major depression [58] and the study of Savitz et al. (2018) in bipolar depression [60]. They lasted eight and six weeks, respectively. These studies, therefore, only assessed the potential efficacy of ASA in treating a depressive episode in the short term and did not address the potential long-term efficacy of ASA in preventing relapse, as suggested by observational studies. Only one intervention study has attempted to evaluate the long-term efficacy of ASA [61]. The study lasted two years and did not confirm the beneficial effect of ASA on major depression. However, it should be noted that this was an open label study with a small group (*n* = 40). Therefore, more long-term double-blind RCTs are needed to assess whether ASA has a beneficial long-term effect on the course of recurrent mood disorders, such as bipolar disorder or recurrent major depressive disorder.

A key aspect of any therapy, especially long-term therapy, is also safety. In none of the studies included in this review did the addition of ASA to therapy result in serious adverse effects or an increase in bleeding events. One of the longest interventional studies, lasting two years, also confirmed its safety [61]. One of the main adverse effects of lithium is nephrotoxicity. Studies report that it may contribute to the development of chronic kidney disease, as well as diabetes [74,75,76]. ASA improved renal parameters in rats treated with lithium and ASA combination therapy, suggesting a protective effect of ASA on kidneys [72]. Sexual dysfunction is another adverse effect of lithium. In euthymic patients, ASA improved sexual function, confirming its benefits also among stable BD patients [57]. This is due to the regulation of prostaglandin production and the NO pathway [77], which is inhibited by lithium intake [78]. Despite some evidence that ASA improves erectile function in patients with vascular erectile dysfunction, the relationship between erectile dysfunction and ASA remains unclear [79,80,81,82]. This requires additional research, long-term evaluation, and association of ASA with other mood stabilizers. There is one more potential benefit of ASA—patients with BD are at increased risk of secondary vascular disease, thus the anticoagulant effect of ASA therapy may reduce their mortality [83,84,85].

It is important to mention that ASA has the potential to cause drug—drug interactions, as with any other substance [86]. Therefore, each patient should be evaluated for somatic conditions and other medications, especially antiplatelet, anticoagulant, or anti-inflammatory drugs. Multiple anticoagulants drug combined can lead to impaired coagulation and gastrointestinal problems [87]. NSAIDs used chronically and at full doses might interfere with a steady 24-h platelet COX-1 inhibition of aspirin. This is particularly important because mood disorders are associated with increased risk of cardiovascular events. A synergic effect of SSRIs and ASA has also been discussed, but no serious hemorrhagic effects were observed in the included studies [88]. As herbal medicine is gaining popularity, it is importance to note herbs can have antiplatelet effects [89]. ASA is unaffected by commonly used adult drugs, such as PPIs, ACEI, ARBs [88]. As a result, although ASA is considered safe, it should be evaluated individually and carefully by a physician before being added to a treatment plan. Nevertheless, we found no evidence in the identified studies to suspect adverse interactions between ASA and medications taken by patients for mood disorders (SSRI drugs, duloxetine, lithium, anticonvulsants, antidepressants, antipsychotics, benzodiazepines). Regarding the potential risk in various somatic conditions and the associated treatment of these conditions, we did not find such data in the identified studies. Thus, this would require further research.

The question of both the efficacy and safety of ASA is closely related to the dose used. We analyzed this starting from preclinical studies, followed by cohort and interventional studies. According to the preclinical trials, when a low dose of ASA (1–50 mg/kg) was used, five out of six studies showed a positive result [63,64,65,66,67]. No improvement in efficacy was found in a study that used a high dose of ASA [68]. Similarly, in large observational studies, the risk of mood disorders decreased with low dose and increased with high dose [39,40]. In the intervention studies, ASA doses ranged from 100 mg/d to 1000 mg/d. However, ASA doses of 162 mg/d and 160 mg/d were found to be effective. It appears that both excessively high (1000 mg/d) and excessively low (100 mg/d) doses of ASA are ineffective. However, it should be noted that ASA is well tolerated at a dose of 100 mg for two years [61], as well as at a very high dose of 1000 mg/d for 16 weeks [59].

**Figure 8:**
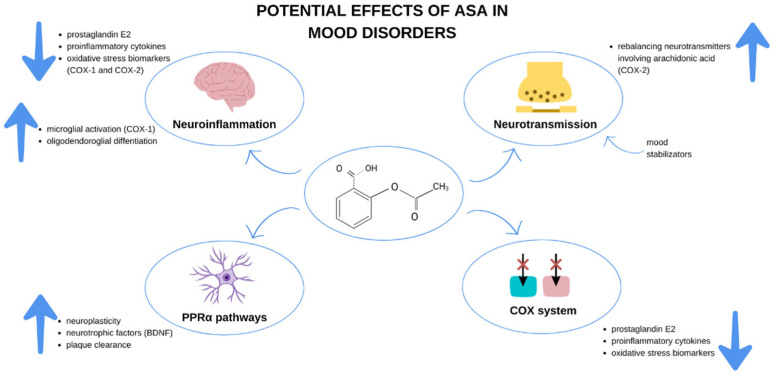
Potential mechanisms of action in mood disorders.

Another issue addressed in this review is the potential mechanism of action of ASA in mood disorders. The potential therapeutic effect of acetylsalicylic acid is likely to be due to its ability to act via COX-1 and COX-2 and its effect on the arachidonic pathway. Several clinical trials have shown that COX-2 inhibition with a selective inhibitor such as celecoxib also improves depressive symptoms in both unipolar and bipolar depression, according to a recent meta-analysis [90]. Despite growing evidence for the efficacy of COX-2 inhibition, COX-1 is also involved in neuroinflammation [41]. Adult microglia express COX-1, and microglia activation contributes to mood disorders [91,92,93]. In addition to influencing the production of inflammatory metabolites, arachidonic acid is also a second messenger released during neurotransmission by various neurotransmitter systems [94]. Therefore, this raises the possibility that inhibition of COX by ASA may modulate impaired neurotransmission in depression. Inflammatory responses are probably mediated by these enzymes (COX-1 and COX-2) together, although further research is needed [95]. Another possible mechanism of action of ASA was investigated in a preclinical study in an animal model [96]. The authors found that ASA may affect the regulation of brain-derived neurotrophic factor (BDNF) in hippocampal neurons. We have visualized these concepts in Figure 8.

During the literature review, we found some evidence in preclinical trials that inflammatory parameters may serve as useful markers in mood disorders. However, although preclinical studies have confirmed that ASA increased anti-inflammatory effects (normalizes levels of IL-6, TNF-alpha, CRP, IL-1β, IL-2, IL-10, INF- γ, PGE2, BDNF, TLR3, TLR4 [66,67,71]), this has not been confirmed in the few clinical studies conducted to date [59,60]. It seems that anti-inflammatory treatment in mood disorders may depend on identifying patients with an inflammatory phenotype, i.e., those whose inflammation contributes to the development of the disorder. This interesting issue would certainly require further clinical studies.

The combination of more than one anti-inflammatory agent is also a promising prospect that needs to be explored further. We identified two studies in which N-acetylcysteine or minocycline were combined with ASA [59,60]. Moreover, in one of them, N-acetylcysteine and ASA were combined to produce a positive effect [59]. The results of some trials examining these anti-inflammatory agents separately were also positive, but they are inconclusive and further research is necessary to determine the benefits of their combination [97,98].

Lastly, it is worth pointing out that this review focused on the adult population (>18 years of age), excluding studies on the elderly population. However, during the literature review, we also identified studies and reviews on the efficacy of ASA in two other patient populations—older adults and adolescents [99,100]. It is worth noting that these are very different populations, responding differently to treatment for mood disorders, mainly due to different disease course, drug pharmacodynamics, and comorbidities. In particular, depression in the elderly population is particularly resistant to treatment. We believe that this is why none of the studies on these populations have produced positive results. Hence, the effect of ASA on mood disorders in these specific populations deserves a separate systematic review. In this review, we analyzed intervention studies in which the range of mean patient age was between 37–48 years, with a mean of 42.5 years. Regarding gender, in all studies, (with the exception of one in which only men participated), the proportion of women was higher—women on average accounted for 68% of the study group. Importantly, however, none of the studies found evidence that age or gender affected the results of efficacy or safety of ASA therapy.

### Limitations

In this review, conclusions are based on a relatively small number of intervention studies and are, therefore, subject to change with the addition of further studies. In addition, most of the studies included small sample sizes and were of short duration, which limits the conclusions regarding the long-term efficacy of ASA in mood disorders. We also included only studies written in English. Due to differences in outcome measures, as well as different study populations (especially in intervention studies), the results could not be pooled and meta-analyzed.

## 5. Conclusions

This systematic review supports the safety and efficacy of low-dose adjunctive treatment with ASA for mood disorders. There is still a need for preclinical and clinical studies to assess the long-term use of ASA and the effectiveness in preventing relapses of affective episodes. In particular, high-quality double-blind RCTs are lacking. The largest research gaps we have identified relate to the assessment of long-term effects on the course of BD, effects on mania in BD, and the investigation of the relationship with inflammatory cytokines and the inflammatory phenotype.

## Figures and Tables

**Table 1:** PICO questions.

	Preclinical Studies	Observational Studies	Interventional Studies
Patients/Subjects	Studies on mania or depression animal models. Animals may or may not be treated with mood stabilizers or antidepressants	Patients receiving chronic low-dose of ASA, with or without a diagnosis of major depression (MD) or bipolar disorder (BD), under 65 years of age and over 18 years of age	Patients diagnosed with BD or MD; under 65 years of age and over 18 years of age
Intervention	Use of ASA alone or as an add-on treatment	Patients receiving chronic low-dose of ASA	Treated in combination with ASA
Comparison	No use of ASA	Patients not chronically taking a low dose of ASA	No use of ASA; placebo
Outcome	Impact on behavioral tests; histological and biochemical analysis	Episodes of depression, mania: hazard ratio (HR); incident density (ID); odds ratio (OR)	Impact on affective episodes (depressive/manic/mixed)–clinical scales; adverse effects

**Table 2:** In Vivo Studies on ASA in Animal Models of Depression or Mania.

Author, Year, Country	Species Sample size (n) Sex (M/F)	Drugs Tested	Procedure	Results
Alboni et al., 2018, Italy	Sprague-Dawley rats n = 5–10/group, M	Fluoxetine 5 mg/kg+ ASA 11.25–45 mg/kg, fluoxetine 5 mg/kg + flurbiprofen 5 mg/kg, fluoxetine 5 mg/kg + celecoxib 5 mg/kg; 7 days of treatment	Chronic escape deficit	Co-administration of ASA with fluoxetine was able to revert the stress induced condition of escape deficit. The effect of ASA was dose dependent
Brunello et al., 2006, Italy	Sprague-Dawley rats n = 7–8/group, M	ASA 45 mg/kg or 22.5 mg/kg, fluoxetine 5 mg/kg	Chronic escape deficit	Fluoxetine alone needs to be administered for three weeks to revert the stress induced escape deficit. Combined treatment of ASA and fluoxetine reverted the condition in seven days. ASA alone was ineffective.
Guan et al., 2014, China	Sprague-Dawley rats n = 7/group, M	ASA 6, 12, 25 and 50 mg/kg subacute treatment	Forced Swimming Test,	ASA dose dependently decreased immobility in FST and diminished FST-induced increase in IL-6 and TNF-α
Shvartsur et al., 2021, Israel	Sprague-Dawley rats n = 16/group, M	ASA IP for 42 days; 1 mg/kg alone or together with Li	Elevated Plus-Maze Test; assessment of toxicity (water consumption, urinary output, plasma Li, creatinine, urea, cystatin c levels; nephrin and podocin levels, renal tubulointerstitial damage, gastric mucosal damage and bleeding	The combination of ASA with LLD-Li enhances hedonic behavior. ASA alone did not increase them. Co-administration of low-dose ASA with low-dose Li mitigates typical renal side-effects of standard-dose Li while retaining the beneficial behavioral effects of this enigmatic cation
Shvartsur et al., 2022, Israel	Sprague-Dawley rats n = 16/group, M	ASA for 42 days; 1 mg/kg alone or together with Li	Amphetamine-induced Hyperactivity Test, Forced Swimming Test, Lipopolysaccharide (LPS)-Treated Rats	Chronic co-treatment of ASA with low-dose Li had no effect on amphetamine-induced hyperactivity, decreased immobility time in FST, attenuated LPS-induced hypothermia and plasma and brain cytokine level elevation.
Wang et al., 2011, China	Sprague-Dawley rats n = 7/group, M	ASA 20 mg/kg in conjunction with fluoxetine 10 mg/kg for 3 weeks	Chronic Unpredictable Mild Stress (CUMS) rats resistant to fluoxetine Forced Swimming Test	20–30% CUMS-induced depressive rats were resistant to fluoxetine. ASA adjunctive treatment showed improved sucrose preference and decreased immobility time in FST, as well as inhibition of COX-2 and PGE2 concentration.
Warner-Schmidt et al., 2011, USA	C57BL/6 mice n = 8–16/group, M	ASA 210 mg/kg, Ibuprofen 70 mg/kg, Naproxen 140 mg/kg	Tail Suspension Test, Forced Swimming Test,	Anti-inflammatory drugs antagonized biochemical and behavioral responses to SSRIs
Zhang et al., 2019, China	Sprague-Dawley rats n not given, M	ASA IG for 1 week; 50 mg/kg	Ouabaine- induced hyperlocomotion	ASA decreased ouabaine induced hyperlocomotion and stereotypic behavior.

**Table 3:** Observational studies on the effect of ASA on mood disorders.

Author, Year, Country	Study Design	Sample Size (n), Control Sample Size, Duration	Characteristics of the Study Group	Conclusions
Kessing et al. 2019a, Denmark	Retrospective study	n = 1,605,365, including n = 315,542-taking low-dose ASA Control group-random sample, 30% of the Danish population (1,710,000) 10 years (2005–2015)	Patients receiving studied drugs (ASA—low (75–150 mg/d) and high dose (>500 mg/d), statins, allopurinol, and NSAIDs, drugs acting through angiotensin Age: median of 57 years	Chronic low-dose ASA (75–150 mg/d), statins, and medications that act via angiotensin were associated with a reduced risk of experiencing an episode of mania, another affective episode in BD, or taking lithium (for low-dose ASA: HR 0.92, 95% CI: 0.89 to 0.95; *p* < 0.001) after adjusting for age, sex, employment status, year, and somatic disorders. High-dose ASA (500 mg/d) and NSAID administration was associated with an increased risk of a depressive episode or antidepressant discharge (for high-dose ASA: HR 1.12, 95% CI: 1.03 to 1.22; *p* < 0.001) after adjusting for age, gender, employment, year, and somatic diseases
Stolk et al. 2010, Netherlands	Retrospective study	n = 5145 10-year observation period (1996–2005)	Patients with at least five lithium prescriptions (presumed BD patient population); Mean age 48 years	Low-dose ASA (up to 80 mg/day) reduced the risk of clinical deterioration in patients taking lithium, as well as the adjusted incidence of deterioration—ID (incidence density) (incident dose increase or drug change)—0.84, 95% CI: 0.75 to 0.94; *p* < 0.05)
Kessing et al. 2019b, Denmark	Retrospective study	n = 1,576,253, including n = 315,542-taking low-dose ASA Control group-random sample, 30% of the Danish population (1,710,000) 10 years (2005–2015)	Patients receiving studied drugs (ASA—low (75–150 mg/d and high dose (>500 mg/d) statins, allopurinol, and NSAIDs, drugs acting through angiotensin Age: median of 65 years	Those receiving chronic low-dose ASA (75–150 mg/d), statins, allopurinol, and drugs acting through angiotensin were associated with a reduced risk of a depressive episode or antidepressant discharge (for low-dose ASA: HR 0.93, 95% CI: 0.93 to 0.94; *p* < 0. 001) after adjusting for age, gender, employment status, year, and somatic disorders. High-dose ASA (500 mg/d) and NSAID intake was associated with an increased risk of a depressive episode or antidepressant discharge (for high-dose ASA: HR 1.03, 95% CI: 1.01 to 1.05; *p* < 0.001) after adjusting for age, gender, employment status, year, and somatic disorders
Pasco et al. 2010, Australia	Study 1: Controlled trial	Study group: n = 104 (taking ASA for an average of 7 years); Control group n = 282 Time period-10 years	Female only, over 50 years old, population derived from the Geelong osteoporosis study	The use of ASA in women with both a positive and negative history of major depression was associated with a lower chance of a major depressive episode (OR 0.18, 95% CI: 0.02 to 1.39) after adjustment for age. ASA dose: no data available
	Study 2: Retrospective cohort	Study group: n = 161 Control group: n = 184; Time period-10 years		Taking ASA and statins in women with a negative prior history of depression was associated with a reduced risk of a depressive episode (HR 0.20, 95% CI: 0.04 to 0.85, *p* = 0.03). ASA dose: no data available
Veronese et al. 2018, USA	Cohort of the larger study	Study group: n = 137 (taking ASA); Control group: n = 4003; 8 years	Population from a multi-center, Osteoarthritis Initiative (OAI) study; Average age: 65 years old	Observation of the occurrence of depression in subjects without a prior diagnosis of depression. ASA use did not reduce the risk of depressive symptoms (HR 1.12; 95% CI: 0.78 to 1.62, *p* = 0.54). ASA dose: no data available
Glaus et al. 2015, Switzerland	Prospective study	Study group: n = 132 (receiving ASA); Control group: n = 1499; Mean follow-up 5.2 years	Female and male patients, Average age 51 years old	ASA administration did not reduce the risk of MDD (HR 1.19; 95% CI: 0.68 to 2.08, *p* > 0.05). The study and control groups differed significantly in age (mean age was higher in the study group) (*p* < 0.0001) and in the prevalence of being overweight, diabetes, hypertension, and heart disease, all of which were significantly more common in the study group (all *p* < 0.0001). ASA dose: no data available
Williams et al. 2016, Australia	Study 1: Controlled trial	Study group: n = 8 (receiving ASA for an average of 8.4 years); Control group: n = 140 5 years (2006–2011)	Male patients, mean age of 50 years, observed for 5 years; population derived from the Geelong osteoporosis study	Observation of major depression in individuals without a prior diagnosis of depression. After adjusting for age and antidepressant use, ASA exposure was associated with a lower chance of depression (OR 0.4, 95% CI: 0.2 to 0.9, *p* = 0.03)
	Study 2: Retrospective cohort	Study group: n = 210 (receiving ASA or statins); Control group: n = 626		Reduced risk of major depression in patients with a history of ASA and statin use (HR 0.55, 95% CI: 0.23 to 1.32, *p* = 0.18). ASA dose: no data available

Note: ASA—acetylsalicylic acid; BD—bipolar affective disorder; MDD—major depressive disorder; NSAID—non-steroidal anti-inflammatory drugs.

**Table 4:** Interventional studies with ASA in bipolar disorder and depression.

Author, Year, Country	Study Design	Sample Size (n), Duration	Characteristics of the Research Group (Mean Age, Female Proportion); Medication; Comorbidities	Conclusions
Saroukhani et al. 2013, Iran	Double-blind, randomized placebo-controlled two-arm trial	n = 32 6 weeks	Male patients with stable BD (euthymia) aged 20–45 treated with lithium; ASA group: (35.9 ± 9.0; 0%) Placebo group: (39.6 ± 9.7; 0%); Medications (lithium); Comorbidities–data not given	The purpose of the study—to evaluate the effect of ASA on sexual dysfunction in patients with BD treated with lithium (1500–1800 mg/day). Safety—patients who received ASA (240 mg/day) reported improvements in overall sexual function scores. Good tolerability of treatment, no differences between groups, no effect of ASA on lithium blood levels.
Bauer et al. 2018, USA	Double-blind, randomized placebo and N-acetylcysteine (NAC)-controlled four-arms trial	n = 25 16 weeks	Patients with bipolar depression aged 16–65 years; ASA group: (49 ± 15.21; 75%) ASA+NAC group: (40 ± 17.64; 25%) NAC group: (36.38 ± 7.05; 62.5%) Placebo group: (39.13 ± 9.99; 75%); Medications (lithium, anticonvulsants, antidepressants, antipsychotics, benzodiazepines), with no signifiant differences between groups; Comorbidities (agoraphobia, GAD, panic disorder, PTSD, OCD, alcohol abuse, substance use, bulimia, BED), with no signifiant differences between groups	Four groups: ASA (1 g/day), NAC (1 g/d), ASA+NAC (1 g/d + 1 g/d), placebo. The probability of response at week 16 was: 0.67 (95% CI, 0.54 to 0.81) for ASA + NAC, 0.57 (95% CI, 0.45 to 0.7) for NAC + placebo, 0.55 (95% CI, 0.44 to 0.67) for placebo and 0.33 (95% CI, 0.2 to 0.45) for ASA + placebo. The ASA group (1 g/d) was not significantly different from the placebo group. Safety—no severe adverse effects were noted
Savitz et al. 2018, USA	Multi-center randomized placebo and minocycline-controlled four-arms trial	n = 99 6 weeks	Patients with moderate bipolar depression, 18–65 years; ASA group: (40.6 ± 10.2; 68%) ASA+minocycline: (40.8 ± 9.7; 84%) Minocycline group: (44.8 ± 8.7; 68%) Placebo group: (40.8 ± 10.4; 73%); Medications (mood stabilizers, antidepressants, antipsychotics, anxiolytics), with no signifiant differences between groups; Comorbidities–data not given	Four groups: ASA (162 mg/day), minocycline (200 mg/d), ASA+placebo (162 mg/d), minocycline+placebo (200 mg/d). There was a significant main effect of ASA on clinical response rates (χ2 = 5.52, *p* = 0.019). The NNT for response rate was 4.2 NNT for achieving remission. 6.5. Safety–no severe adverse effects were noted
Sepehrmanesh et al. 2017, Iran	Double-blind, randomized placebo-controlled two-arm trial	n = 100 8 weeks	Patients with major depression; ASA group: (48.9 ± 7.5; 58%) Placebo group: (47.8 ± 7.3; 64%); Medications (sertraline); Comorbidities (anxiety), with no signifiant differences between groups;	Two groups: patients treated with sertraline (50–200 mg/day)+ASA (dose 160 mg/d) and patients treated with sertraline+placebo. Patients in the group taking additional ASA (160 mg/d) had a greater reduction in depressive symptoms after eight weeks compared to the placebo group (*p* < 0.008). Safety—side effects among patients who received ASA were as similar as patients who received placebo
Zdanowicz et al. 2017, Belgium	Open label, randomized placebo-controlled four-arms trial	n = 40 2 years	Patients with major depression, aged 18–63; ASA group: (46.4; 82.5% in all patients) Placebo group: (34.25; 82.5% in all patients); Medications (duloxetine, escitalopram); Comorbidities–data not given	Four groups: ASA (100 mg/day), +duloxetine, duloxetine+placebo, escitalopram + ASA (100 mg/day), escitalopram + placebo. No difference was observed between groups taking ASA and placebo. A difference was found in additional subgroup analyses—between the duloxetine+ASA and escitalopram+placebo groups—faster reduction of depressive symptoms on the HAMD scale in the duloxetine+ASA group (t = −3.114, *p* = 0.01) and higher remission rates (chi^2^ = 6.296, *p* = 0.012). Safety—no report about adverse effects
Mendlewicz et al. 2006, Belgium	Clinical trial: Pilot open-label study	n = 21 4 weeks	24 patients with bipolar (n = 4) or unipolar depression (n =17), treated for four weeks with SSRIs without response to treatment, aged 29–62; ASA group: (46.1±9.7; 61.9%); Medications—various SSRI drugs were used as the main treatment (citalopram, fluoxetine, paroxetine, sertraline, fluvoxamine, escitalopram), as well as mood stabilizers Comorbidities–data not given	ASA (dose 160 mg/d) was added to the SSRI treatment. Response to treatment was achieved in 47% of patients with unipolar and 75% of patients with bipolar depression, and remission was achieved in 41% of patients with unipolar and 50% of patients with bipolar depression (measured using the HAMD-21 scale). The observed improvement occurred after one week of treatment with added ASA (HAMD baseline=29.3 ± 4.5, at day 7 = 14.0 ± 4.1; *p* < 0.0001); Safety–no report about adverse effects

Note: HAMD—Hamilton Depression Rating Scale, ASA—acetylsalicylic acid; NNT—number needed to treat; SSRI—selective serotonin reuptake inhibitor; NAC—N-acetylcysteine, BED–binge eating disorder; GAD–generalized anxiety disorder; OCD–obsessive-compulsive disorder; PTSD–posttraumatic stress disorder.

## Data Availability

The data presented in this study are available on request from the corresponding author. The data are not publicly available due to privacy or ethical concerns.
